# Impact of diabetes on hospital admission and length of stay among a general population aged 45 year or more: a record linkage study

**DOI:** 10.1186/s12913-014-0666-2

**Published:** 2015-01-22

**Authors:** Elizabeth Jean Comino, Mark Fort Harris, MD Fakhrul Islam, Duong Thuy Tran, Bin Jalaludin, Louisa Jorm, Jeff Flack, Marion Haas

**Affiliations:** Centre for Primary Health Care and Equity, University of New South Wales, Sydney, NSW 2052 Australia; Centre for Big Data Research in Health, University of New South Wales, Sydney, NSW 2052 Australia; Centre for Research, Evidence Management and Surveillance, Sydney and South Western Sydney Local Health Districts, Locked Bag 7017, Liverpool, NSW 1871 Australia; School of Public Health and Community Medicine, University of New South Wales, Sydney, 2052 Australia; Centre for Health Research, School of Medicine, University of Western Sydney, Locked Bag 1797, Penrith, NSW 2751 Australia; Bankstown-Lidcombe Hospital, Eldridge Road, Bankstown, NSW 2200 Australia; Centre for Health Economics Research and Evaluation, Faculty of Business, University of Technology, Sydney, PO Box 123, Broadway, NSW 2007, Level 4, 645 Harris Street, Ultimo, NSW 2007 Australia

**Keywords:** Healthy aging, Diabetes, Hospital admission, Record linkage, Cohort study, Socioeconomic status, Health and wellbeing

## Abstract

**Background:**

The increased prevalence of diabetes and its significant impact on use of health care services, particularly hospitals, is a concern for health planners. This paper explores the risk factors for all-cause hospitalisation and the excess risk due to diabetes in a large sample of older Australians.

**Methods:**

The study population was 263,482 participants in the 45 and Up Study. The data assessed were linked records of hospital admissions in the 12 months following completion of a baseline questionnaire. All cause and ambulatory care sensitive admission rates and length of stay were examined. The associations between demographic characteristics, socioeconomic status, lifestyle factors, and health and wellbeing and risk of hospitalisation were explored using zero inflated Poisson (ZIP) regression models adjusting for age and gender. The ratios of adjusted relative rates and 95% confidence intervals were calculated to determine the excess risk due to diabetes.

**Results:**

Prevalence of diabetes was 9.0% (n = 23,779). Age adjusted admission rates for all-cause hospitalisation were 631.3 and 454.8 per 1,000 participant years and the mean length of stay was 8.2 and 7.1 days respectively for participants with and without diabetes. In people with and without diabetes, the risk of hospitalisation was associated with age, gender, household income, smoking, BMI, physical activity, and health and wellbeing. However, the increased risk of hospitalisation was attenuated for participants with diabetes who were older, obese, or had hypertension or hyperlipidaemia and enhanced for those participants with diabetes who were male, on low income, current smokers or who had anxiety or depression.

**Conclusions:**

This study is one of the few studies published to explore the impact of diabetes on hospitalisation in a large non-clinical population, the 45 and Up Study. The attenuation of risk associated with some factors is likely to be due to correlation between diabetes and factors such as age and obesity. The increased risk in association with other factors such as gender and low income in participants with diabetes is likely to be due to their synergistic influence on health status and the way services are accessed.

## Background

Diabetes mellitus is a serious public health problem that has implications for individuals, communities, and health and human services [1]. The rapid increase in the prevalence of diabetes, driven by the increased prevalence of obesity and an aging population, has led to it being described as an epidemic. Diabetes comprises a complex of metabolic disorders associated with impaired insulin secretion and glucose metabolism [2,3]. Among older people, diabetes is associated with poor health outcomes including hyperglycaemia, increased cardiovascular risk, and peripheral vascular problems, and is associated with increased use of health services including unplanned hospitalisation and premature mortality [3,4]. The importance of early detection and management of diabetes to prevent disease progression, poor health outcomes including early onset of complications, and increased use of health services is recognised and supported by policy and practice interventions to improve diabetes care. Yet diabetes remains a significant reason for preventable contact with the health system [5,6].

A number of studies have demonstrated that people with diabetes [5,7-10] have hospital admission rates between 2 and 6 times higher than people without diabetes [5,9-11]. People with diabetes also have excessive lengths of hospital stay compared to people without diabetes [5,9,11]. These previous studies used hospital or practice-based populations [5,9,11]. Study of hospital-based populations may represent people with severe diabetes including complications of diabetes and its associated morbidity. As results, the associated risks as well as hospitalisation rates could be over-estimated. There is a need to determine the risk of hospitalisation and impact of diabetes among a general community population.

It is generally accepted that risk factors such as age, gender, education, socioeconomic status, lifestyle risk factors, and health status are associated with poorer health outcomes including hospitalisation among the general population. This is no less so for people with diabetes where lower socioeconomic status, older age, obesity, tobacco smoking, physical inactivity, poor glycaemic control and clinical indicators including glycosylated haemoglobin (HbA1c), insulin use, longer duration of diabetes, and presence of complications have been associated with increased rates of hospitalisation and longer length of stay [7,12,13]. Few studies have examined these associations for a community dwelling population of people with and without diabetes.

The establishment of a large population based cohort of New South Wales, Australia residents aged 45 year and older at recruitment and enhanced linkage facilities enabled us to create a community dwelling population study of diabetes. In this paper we describe the association between hospital admission and wide range of demographic characteristics, socioeconomic status, lifestyle, and health and wellbeing among study participants with and without diabetes. We also explore the excess risk of hospitalisation that may be attributed to diabetes.

## Methods

Data were obtained from the following sources:

### The Sax institute: 45 and Up study

The Sax Institute’s 45 and Up Study is a cohort study of more than 250,000 residents of NSW, Australia. Recruitment of this cohort has been described elsewhere [14]. Briefly, potential study participants comprised progressive random samples of adults aged 45 years or older registered on the Medicare Australia (a universal health insurance scheme) database held by the Department of Human Services. Participants joined the Study by completing a baseline questionnaire and providing consent for long-term follow up including linkage of their baseline data to their health records. Recruitment was undertaken between February 2006 and April 2009.

The baseline questionnaire collected information on a range of participant characteristics (available at www.45andup.org.au). Our method of identifying participants with diabetes at baseline is described elsewhere [15]. The majority of participants was defined as having diabetes based on their response to Question 24: ‘*Has a doctor EVER told you that you have diabetes*?’. Other diabetic participants were identified on the basis of free-text responses to a question eliciting current important illnesses and self-reported use of diabetes medications. The baseline survey did not differentiate type of diabetes. We included all participants identified as having diabetes.

Demographic variables (age, date of recruitment, gender, country of birth), socioeconomic status (highest education level, household income), and lifestyle (smoking status, alcohol intake, body mass index (BMI), and physical activity) were obtained from the baseline questionnaire. BMI (Kg/M^2^) was classified as normal weight (<25), overweight (25–29), and obese (>30). Physical activity was classified as sedentary if no physical activity was reported and sufficient if physical activity comprised at least 150 minutes of walking during at least 5 sessions per week; all other categories were classified as insufficient [16].

Socioeconomic measures of residential areas included the Australian Bureau of Statistics Index of Relative Socioeconomic Disadvantage (SEIFA IRSD) [17] and remoteness according to the mean Accessibility Remoteness Index of Australia Plus (ARIA+) score for the Statistical Local Area of residence [18]. SEIFA IRSD was classified as quintile where 1 was the least and 5 the most disadvantaged. ARIA+ was classified as major city, inner regional, and outer regional and remote (grouped). Health status was measured in a number of ways. Self-rated general health and quality of life were self-rated as excellent or very good, good, and fair or poor. Psychological distress based on the Kessler-10 score [19] was categorised as low (score of 10–15), moderate (16–21), high (22–29) and very high (30–50). Functional capacity was measured using the Medical Outcomes Study, Short Form 36 Physical Functioning Scale (SF36-PF) [20] and classified as no limitation (score of 100), minor (90–99), moderate (60–89) and severe (0–59). The choice of cut-off for these scores was based on previous research [21]. The number of chronic conditions was identified from participants’ responses to the questions “*Has a doctor ever told you that you have* . . . .” or ‘*In the last month have you been treated for* -?’ and listed a number of chronic health conditions including cancer, heart disease, high blood pressure, stroke, anxiety, and depression. Participant responses were summed and classified as none, 1, 2, and 3 or more. Among participants with diabetes, duration of diabetes was calculated from age at diagnosis and age at questionnaire completion.

### NSW ministry of health: admitted patient data collection

Under the Australian health care system, hospital services in New South Wales are provided through a mix of publicly and privately funded health services. The NSW Ministry of Health has responsibility for all inpatient services and collates data on admissions into the NSW Admitted Patient Data Collection (APDC). Data were available for 2000–2009. The APDC collates inpatient admissions (discharges, transfers and deaths) from all public, private, and repatriation hospitals, private day procedure centres and public nursing homes in NSW. These data include demographic characteristics, diagnoses, and length of stay for individual episodes of hospitalisation. The diagnoses were coded using International Classification of Disease 10th revision-Australian Modification (ICD-10-AM) codes. APDC data were available for this study for 2000–2009. For these analyses, APDC records were extracted for the 12 months following recruitment for each participant.

### NSW registry of births, death and marriages: death registrations

All deaths in NSW are certified as to cause and date by a registered medical practitioner and the certificate registered by the NSW Registry of Births, Death and Marriages (RBDM). These linked data were used to identify participants who died within 12 months of recruitment to the 45 and Up Study.

### Record linkage

Baseline questionnaire data from the 45 and Up study were linked to APDC data for the 12 month period following recruitment to the study and to RBDM to exclude participants who dies during this 12 month period. Record linkage was undertaken by the Centre for Health Record Linkage (CHeReL) according to privacy-preserving protocols and probabilistic methods [22,23]. Linked records were assigned a unique ‘project person number’ which was then returned to the data custodians who released de-identified clinical data to the research team.

Of the 266,771 participants in the 45 and Up Study with baseline information, we excluded 3,289 participants due to linkage errors (n = 24), death within 12 months of recruitment (n = 1,632), admission for dialysis (n = 251), and uncertain diabetes status (n = 1,382). The final dataset for analysis contained baseline 45 and Up Study data and hospital admission information for 263,482 participants, of whom 23,779 (9.0%) had diabetes and included 124,035 hospitalisation episodes within 12 months of recruitment, involving 65,777 participants.

### Outcome measures

The primary outcome for this study was any hospitalisation within 12 months following recruitment to the 45 and Up Study. Firstly, participants were categorised as having at least one hospital admission or no hospital admission, and secondly, the aged standardised hospital admission rates (rate of admission per 1,000 participants) were calculated. In addition we estimated for the 12 month period the mean number of hospital admissions, total number of days, and length of stay among participants with and without diabetes.

Using the ICD-10-AM codes, the diagnostic reasons for hospitalisation were categorised as all cause, diabetes where diabetes (E10-E14.9) was the principal reason for admission, metabolic or cardiovascular disease (MCVD), and diabetes related ambulatory care sensitive conditions (ACSCs) [24]. MCVD diagnosis as the principal reason for admission included conditions that were considered to be complications of diabetes (hypersmolarity (E87.0) acidosis (E87.2), transient ischaemic attack (G45), nerve disorders and neuropathies (G50–G64), cataracts and lens disorders (H25–H28), retinal disorders (H30–H36), glaucoma (H40–H42), myocardial infarction (I21–I22), other coronary heart diseases (I20, I23–I25), heart failure (I50), stroke and sequelae (I60–I64, I69.0–I69.4), peripheral vascular disease (I70–I74), gingivitis and periodontal disease (K05), kidney diseases (N00–N29) including end-stage renal disease (N17-N19), and renal dialysis (Z49)). Diabetes related ACSCs were those admissions where principal diagnosis was diabetes or the principal diagnosis was an MCVD code (listed above) and diabetes was given as an additional diagnosis [24]. Length of stay (days) for each admission was calculated from the dates of admission and discharge, taking into account transfers to other facilities during the hospital stay. We calculated the total number of days of hospitalisation during the 12 months and the mean length of stay for each admission.

### Statistical analysis

Descriptive univariate and multivariate methods were used in these analyses. Measures of hospitalisation took into account transfers and same day readmissions. In order to handle the skewed data, we built zero inflated Poisson (ZIP) regression models with a log link function to account for a high proportion of participants who were not admitted during the study period [25]. Adjusted relative rates (aRRs) of hospitalisation (rate per participant year of follow up) with 95% confidence intervals (95% CI) were estimated from univariate and multivariate analyses respectively. All multivariate models were adjusted for age and gender. Missing data for each study factor were included in the multivariate model using ‘missing values’ categories; no variables were excluded due to missing values. We explored the changes in observed associations for hospital admission for all cause, ACSC, and non-elective admissions. It was resolved to use all cause admissions for this study.

Significant interactions were observed between diabetes status and hospitalisation due to the presence of many of the participant characteristics. Consequently, separate models for participants with and without diabetes were built. In order to compare the effect size of the observed associations between participants with and without diabetes, the ratios of adjusted relative rates (Ratio of RR) and their associated 95% confidence intervals were calculated [26]. A ratio of RR of 1.0 indicates that there is no difference between people with or without diabetes in the strength of the association between the participant characteristic and hospitalisation whereas a value greater than or less than 1.0 indicates enhancement or attenuation of the association between the participant characteristic and hospitalisation. All analyses were carried out in SAS version 9.3 (SAS Institute Inc., Cary, NC, USA). All the tests were two-sided and a p-value of less than or equal to 0.05 was considered statistically significant.

### Ethics

The 45 and Up Study was approved by the University of New South Wales Human Research Ethics Committee. The study was approved by the New South Wales Population and Health Services Research Ethics Committee (HREC/09/CIPHS/2, 12 May 2009).

## Results

### Study sample

The participant characteristics (n = 263,482) by diabetes status are summarised in Table 1. Participants with diabetes were more likely to be male, aged 65–74 years, born overseas, have not completed year 10 of school, live in a disadvantaged suburb, and to report a household income of less than $20,000 compared to participants without diabetes. Participants with diabetes were less likely to drink alcohol but more likely to be ex-smokers, obese, physically inactive, and have heart disease, high blood pressure, high blood cholesterol, depression and anxiety compared to those without diabetes.

**Table 1:** **Demographic**, **socioeconomic**, **lifestyle**, **and health and wellbeing characteristics of participants stratified by diabetes status**

**Demographic characteristics**	**Diabetes**		**No diabetes**		**p**-**value**
	**n =23,779**	**%**	**n = 239,703**	**%**	
**Gender**					<0.001
Male	13,393	56.3	108,589	45.3	
Female	10,386	43.7	131,114	54.7	
**Age group** **(years)**					<0.001
45-59	6,698	28.2	115,733	48.3	
60-74	11,143	46.9	86,102	35.9	
≥75	5,935	25.0	37,850	15.8	
**Duration of diabetes** **(years)**					<0.001
<5	7,205	30.3	N/A		
5-9	5,670	23.8	N/A		
10-14	3,933	16.5	N/A		
≥15	4,914	20.7	N/A		
**Country of birth**					<0.001
Australia	17,020	71.6	180,474	75.3	
Overseas	6,759	28.4	59,229	24.7	
**Socioeconomic status**					
**Education**					<0.001
University	3,688	15.5	57,312	23.9	
Trade/Certificate/Diploma	7,178	30.2	76,648	32.0	
At least year 10	7,964	33.5	75,707	31.6	
Less than Year 10	4,379	18.4	26,302	11.0	
**Household income** **($AUD)**					<0.001
≥$70,000	2,965	12.5	59,4166	24.8	
$40,000-$69,999	3,191	13.4	43,588	18.2	
$20,000-$39,999	4,536	19.1	41,579	17.4	
<$20,000	7,585	31.9	43,937	18.3	
**SEIFA IRSD** **(quintiles)**					<0.001
(least disadvantaged) 1^st^	3,597	15.1	51,117	21.3	
2^nd^	4,148	17.4	44,285	18.5	
3^rd^	6,633	27.9	63,680	26.6	
4^th^	5,778	24.3	54,034	22.5	
(most disadvantaged) 5^th^	3,611	15.2	26,383	11.0	
**ARIA+**					0.2
Major City	10,769	45.3	107,637	44.9	
Inner Regional	8,335	35.1	84,351	35.2	
Outer regional/Remote	4,665	19.6	47,524	19.8	
**Lifestyle risk factors**					
**Tobacco smoking status**					<0.001
Never smoked	11,833	49.8	136,842	57.1	
Ex-smoker	10,219	43.0	84,866	35.4	
Current	1,726	7.3	17,987	7.5	
**Alcohol consumption** **(standard drinks/** **week)**					<0.001
0	11,073	46.6	74,732	31.2	
1-6	5,783	24.3	69,818	29.1	
7-13	2,947	12.4	45,951	19.2	
14-20	1,846	7.8	26,881	11.2	
≥20	1,445	6.1	18,308	7.6	
**BMI** **(kg/** **m** ^**2**^ **)**					<0.001
Normal weight (<25)	4,546	19.1	88,806	37.1	
Overweight (25–29)	8,083	34.0	88,255	36.8	
Obese (≥30)	9,406	39.6	47,087	19.6	
**Physical activity** **					<0.001
Sedentary	3,084	13.0	18,191	7.6	
Insufficient	5,043	21.2	41,679	17.4	
Sufficient	13,459	56.6	165,857	69.2	
**Health status**					
**Number of chronic conditions**					<0.001
**0**	9,788	41.2	131,208	54.7	
**1**	7,632	32.1	68,868	28.7	
**2**	3743	15.7	28,131	11.7	
≥**3**	2616	11.0	11,496	4.8	
**Heart disease**					<0.001
No	18,228	76.7	214,275	89.4	
Yes	5,551	23.3	25,428	10.6	
**High blood pressure**					<0.001
No	13,092	55.1	186,431	77.8	
Yes	10,687	45.0	53,272	22.2	
**Hyperlipidaemia**					<0.001
No	16,267	68.4	207,375	86.5	
Yes	7,512	31.6	32,328	13.5	
**Depression**					<0.001
No	16,720	70.3	174,994	73.0	
Yes	4,262	17.9	33,874	14.1	
**Anxiety**					<0.001
No	18,248	76.7	185,358	77.3	
Yes	2,734	11.5	23,510	9.8	
**Wellbeing**					
**SF36** **(level of limitation)**					<0.001
No (100)	3,643	15.3	75,231	31.4	
Minor (90–99)	4,615	19.4	61,299	25.6	
Moderate (60–89)	6,385	26.8	50,387	21.0	
Severe (0–59)	6,866	28.9	29,032	12.1	
**K**-**10** **(level of psychological distress)**					<0.001
Low (10–15)	13,934	58.6	165,517	69.1	
Moderate (11–21)	3,491	14.7	33,362	13.9	
High (22–29)	1,505	6.3	11,063	4.6	
Very high (30–50)	797	3.4	4,307	1.8	
**Self**-**rated general health**					<0.001
Excellent/Very good	5,910	24.9	127,369	53.1	
Good	9,638	40.5	76,297	31.8	
Fair/poor	7,422	31.2	27,847	11.6	
**Self**-**rated quality of life**					<0.001
Excellent/Very good	9,082	38.2	144,058	60.1	
Good	8,482	35.7	62,099	25.9	
Fair/poor	4,820	20.7	21,187	8.8	

Note: Percentages do not consistently total to 100% due to missing values.

### Impact of diabetes on hospital admission

Table 2 summarises the hospital admission data. There were 124,035 admissions among 65,777 participants (25.0%) who had at least one hospital admission recorded in the 12 months following their recruitment; participants with diabetes were more likely to have at least one admission (32.8%) than participants without diabetes (24.2%).

**Table 2:** **Summary of hospital admission and length of stay stratified by diabetes status**

	**Diabetes**	**No diabetes**
**Number of participants**	**N =** **23,** **779**	**N =** **239,** **703**
**Number of admissions**		
Total admissions	16,692	107,343
day only	10,231	75,197
>1 day	6,460	32,145
**Number (%)** **of participants admitted**		
All-cause	7,807 (32.8)	57,970 (24.2)
MCVD^1^ events	1,034 (4.3)	7,752 (3.2)
Diabetes as principal cause	710 (3.0)	-
Diabetes ACSC^2^	1,744 (7.3)	-
**Rate of hospital admission** **(age standardised rate per 1,** **000 participant years)**		
All-cause	631.3 (624.9-637.7)	454.8 (453.0-456.6)
MCVD events	86.8 (83.2-90.3)	65.8 (64.9-66.8)
Diabetes as principal cause	49.8 (47.0-52.5)	-
Diabetes ACSC	136.5 (132.2-140.9)	-
**Rate of hospital admission per participant**		
**Number of hospital days for all** **-cause admissions,** **including day only admissions**		
All-cause		
Number of participants	7,807	57,970
Mean (SD)	8.3 (18.6)	5.5 (12.4)
Median (Min - Max)	2 (1–472)	1 (1–476)
Interquartile range	1-8	1-4
**Length of stay** **(days)** **for all-** **cause admissions,** **excluding day only admissions**		
All-cause		
Number of participants	2,826	15,787
Mean (SD)	8.2 (12.2)	7.1 (9.9)
Median (Min , Max)	4.8 (1.2 - 220)	4 (1.1 - 241)
Interquartile range	3 - 8.6	2.5 - 7.7

^1^MCVD: metabolic or cardio-vascular event in principal diagnostic categories.

^2^ACSC: ambulatory care sensitive condition.

Participants with diabetes (32.8%) were more likely to have a hospitalisation than participants without diabetes (24.2%; aRR: 1.24, 95% CI: 1.21, 1.26). The age adjusted admission rates for all-cause hospitalisation for participants with and without diabetes were 631.3 and 454.8 per 1,000 participant year respectively (Table 2). The number of hospital days among participants with diabetes (mean (SD): 8.3 (18.6) days, median (min-max): 2 (1 – 472) days) were more than those among participants without diabetes (mean (SD): 5.5 (12.4) days, median (min-max): 1 (1 – 476) day) (Table 2). The majority of admissions among those with and without diabetes (61.3% and 70.1% respectively) were day only events. Of participants with an admission, 72% had only one admission. The mean length of stay for admissions of more than one day was 8.2 days (median: 4.8 days) for participants with diabetes and 7.1 days (median: 4 days) for those without diabetes.

The results of regression analyses are presented by diabetes status (Table 3). For participants with and without diabetes, the RR of hospitalisation was higher for male, older age group, Australian-born, lower income, and urban-dwelling participants. Participants living in disadvantaged regions were less likely to have a hospital admission record. Of the lifestyle factors, smoking and sedentary lifestyle were associated with a higher risk of hospitalisation while self-report of higher alcohol consumption was associated with a lower risk. Obesity was associated with hospitalisation for participants with and without diabetes. Health status using both individual and composite measures was also significantly associated with hospitalization for participants with and without diabetes (Table 3).

**Table 3:** **Proportion of participants with a hospital admission**, **and adjusted relative rates** (**rate per participant year**) **and ratios of relative rates of hospital admission** (**all cause**) **by demographic**, **socioeconomic**, **lifestyle**, **and health and wellbeing characteristics among participants with and without diabetes**

**Characteristic**	**Diabetes**		**No diabetes**		**Comparison of aRRs**	
	**% admitted**	aRR* (95% CI)	**% admitted**	aRR* (95% CI)	**Ratio of RR (95% CI)**	**p-value**
**Demographic characteristics**						
**Gender**						
Female	31.5	1	22.9	1	1	
Male	33.9	1.16 (1.12-1.21)	25.8	1.04 (1-02-1-05)	1.12 (1.07-1.16)	<0.001
**Age group** **(years)**						
45-59	23.8	1	17.2	1	1	
60-74	32.5	1.45(1.37-1.53)	26.9	1.65(1.62-1.68)	0.88(0.83-0.93)	<0.001
≥75	43.7	1.72(1.63-1.82)	39.2	2.31(2.27-2.36)	0.74(0.70-0.79)	<0.001
**Country of birth**						
Australia	33.8	1	24.6	1	1	
Overseas	30.3	0.91(0.87-0.95)	22.9	0.94(0.92-0.95)	0.97(0.92-1.01)	0.2
**Socioeconomic status**						
**Education**						
University	29.0	1	20.9	1	1	
Trade/Cert/Diploma	32.9	0.97(0.91-1.03)	24.0	1.00(0.98-1.02)	0.97(0.91-1.04)	0.4
At least year 10	32.3	0.89(0.84-0.95)	24.9	0.98(0.96-1.00)	0.91(0.85-0.97)	<0.001
Less than Year 10	36.1	0.93(0.87-0.99)	29.0	1.02(0.99-1.04)	0.91(0.85-0.98)	0.01
**Household income** **($AUD)**						
$70,000+	25.5	1	19.5	1	1	
$40,000-$69,999	27.4	1.07(0.98-1.17)	21.4	1.02(1.00-1.05)	1.05(0.96-1.15)	0.3
$20,000-$39,999	33.2	1.10(1.01-1.19)	25.4	1.02(0.99-1.05)	1.08(0.99-1.18)	0.09
<$20,000	35.5	1.17(1.08-1.26)	29.0	1.04(1.02-1.07)	1.13(1.04-1.22)	<0.001
**ARIA**+						
Major Cities	33.5	1	25.3	1	1	
Inner Regional	32.8	0.90(0.87-0.94)	23.6	0.85(0.84-0.87)	1.06(1.01-1.10)	0.01
Outer regional/Remote	31.3	0.86(0.82-0.91)	22.7	0.77(0.76-0.79)	1.12(1.06-1.18)	<0.001
**SEIFA IRSD** **(quintiles)**						
(least disadvantaged) 1	35.9	1	26.4	1	1	
2	31.6	0.76(0.71-0.81)	23.1	0.87(0.85-0.89)	0.87(0.81-0.94)	<0.001
3	32.4	0.85(0.80-0.90)	24.1	0.81(0.80-0.83)	1.05(0.99-1.12)	0.1
4	32.7	0.78(0.74-0.83)	23.3	0.77(0.75-0.79)	1.01(0.95-1.08)	0.7
(most disadvantaged) 5	32.2	0.83(0.77-0.88)	23.9	0.80(0.78-0.82)	1.04(0.97-1.11)	0.3
**Lifestyle risk factors**						
**Current tobacco smoking status**						
Never smoked	30.9	1	23.2	1	1	
Ex-smoker	35.6	1.14(1.10-1.19)	26.5	1.13(1.11-1.14)	1.01(0.96-1.05)	0.4
Current	29.6	1.16(1.07-1.25)	20.5	1.02(0.98-1.05)	1.14(1.04-1.24)	<0.001
**Alcohol** **(standard drinks/** **week)**						
0	33.6	1	25.6	1	1	
1-6	31.5	0.91(0.87-0.96)	22.9	0.96(0.94-0.97)	0.95(0.90-1.00)	0.04
7-13	32.2	0.85(0.80-0.91)	23.4	0.90(0.88-0.92)	0.94(0.88-1.01)	0.11
14-20	33.6	0.93(0.86-1.00)	24.6	0.94(0.92-0.96)	0.99(0.91-1.07)	0.8
≥20	30.5	0.89(0.82-0.97)	23.8	0.92(0.89-0.95)	0.97(0.88-1.06)	0.5
**BMI** **(kg/** **m** ^**2**^)						
Normal weight (<25)	34.1	1	22.9	1	1	
Over weight (25–29)	32.5	0.91(0.86-0.95)	24.3	1.05(1.03-1.07)	0.87(0.82-0.91)	<0.001
Obese (≥30)	32.2	1.06(1.01-1.12)	25.8	1.25(1.22-1.27)	0.85(0.80-0.90)	<0.001
Physical activity**						
Sufficient	30.0	1	22.6	1	1	
Insufficient	33.5	1.17(1.11-1.22)	25.8	1.20(1.17-1.22)	0.98(0.93-1.03)	0.3
Sedentary	41.9	1.38(1.31-1.46)	31.7	1.36(1.33-1.39)	1.01(0.96-1.08)	0.6
**Health status**						
**Number of chronic conditions**						
None	24.9	1	19.9	1	1	
1	35.6	1.32(1.26-1.38)	27.1	1.30(1.27-1.32)	1.02(0.97-1.07)	0.5
2	40.0	1.56(1.48-1.65)	31.2	1.55(1.52-1.58)	1.01(0.95-1.07)	0.8
≥3	44.4	1.94(1.84-2.06)	38.0	1.85(1.80-1.90)	1.05(0.99-1.12)	0.1
**Heart disease**						
No	29.7	1	22.6	1	1	
Yes	43.0	1.34(1.29-1.40)	37.2	1.29(1.26-1.31)	1.04(0.99-1.09)	0.10
**High blood pressure**						
No	31.7	1	22.5	1	1	
Yes	34.2	1.05(1.01-1.09)	29.9	1.14(1.12-1.16)	0.92(0.88-0.96)	<0.001
**Hyperlipidaemia**						
No	32.8	1	23.4	1	1	
Yes	32.9	1.03(0.99-1.07)	29.1	1.10(1.08-1.12)	0.94(0.90-0.98)	<0.001
**Depression**						
No	31.7	1	23.8	1	1	
Yes	36.7	1.43(1.37-1.50)	26.9	1.36(1.33-1.38)	1.05(1.00-1.10)	0.04
**Anxiety**						
No	32.1	1	24.0	1	1	
Yes	36.6	1.45(1.38-1.53)	27.1	1.33(1.30-1.36)	1.09(1.03-1.15)	<0.001
**Wellbeing**						
**SF36** **(level of limitation)**						
No (100)	22.0	1	16.8	1	1	
Minor (90–99)	25.3	1.12(1.03-1.22)	21.0	1.25(1.22-1.28)	0.90(0.82-0.98)	0.01
Moderate (60–89)	32.7	1.67(1.55-1.80)	28.9	1.76(1.72-1.80)	0.95(0.88-1.03)	0.2
Severe (0.59)	43.8	2.31(2.15-2.49)	39.9	2.43(2.37-2.49)	0.95(0.88-1.03)	0.2
**K**-**10** **(level of distress)**						
Low (10–15)	30.2	1	22.5	1	1	
Moderate (16–21)	34.8	1.21(1.15-1.28)	24.6	1.22(1.19-1.25)	0.99(0.94-1.05)	0.8
High (22–29)	35.3	1.49(1.38-1.60)	26.8	1.39(1.35-1.44)	1.07(0.99-1.16)	0.09
Very high (30–50)	39.0	1.99(1.83-2.16)	31.2	1.83(1.75-1.91)	1.09(0.99-1.19)	0.08
**Self**-**rated general health**						
Excellent/Very good	24.9	1	19.6	1	1	
Good	31.2	1.35(1.27-1.43)	26.7	1.40(1.37-1.42)	0.96(0.91-1.02)	0.2
Fair/poor	40.6	1.98(1.87-2.10)	36.8	1.95(1.91-1.99)	1.02(0.96-1.08)	0.6
**Self**-**rated quality of life**						
Excellent/Very good	27.3	1	20.9	1	1	
Good	33.1	1.28(1.22-1.34)	27.5	1.27(1.25-1.29)	1.01(0.96-1.06)	0.8
Fair/poor	41.3	1.59(1.52-1.68)	34.8	1.69(1.65-1.73)	0.94(0.89-0.99)	0.03

*Adjusted for age and gender;

**Physical activity: Sufficient: > = 150 minutes of walking and > =5 sessions per week; Sedentary: no physical activity reported; and Insufficient: all other categories.

The adjusted rate ratios for participants with and without diabetes differed significantly for a number of study factors (Table 3). The associations between age, obesity, high blood pressure and high blood cholesterol and hospitalisation were attenuated in people with diabetes compared to those without diabetes. For example, the aRR for age and obesity was lower among participants with diabetes than those without diabetes (Ratio of RR: 0.74, 95% CI: 0.70 – 0.79 and 0.85, 95%CI: 0.80 – 0.90 respectively). In contrast, the association between male gender, low income, current tobacco smoking or having anxiety or depression and hospitalisation was stronger in those with diabetes than in those without diabetes. For example, the aRR for gender, current smokers, and self-report of treatment for anxiety was higher among participants with diabetes than those without diabetes (Ratio of RR: 1.12, 95% CI: 1.07 – 1.16; 1.14, 95% CI: 1.04 – 1.24; and 1.09, 95% CI: 1.03 – 1.15 respectively). Interestingly, the associations between the measures of wellbeing such as K10 did not differ significantly between the two groups.

## Discussion

Our study is one of the largest conducted to date to describe the associations between demographic characteristics, socioeconomic status, lifestyle, and health and wellbeing and hospitalisation among community dwelling population with and without diabetes. Compared to those without diabetes, participants with diabetes were more likely to be male, older, born overseas, have lower educational attainment and income, have health risk factors including obesity and physical inactivity, and have poorer health including chronic conditions, hypertension and hyperlipidaemia, and poorer wellbeing. These participant characteristics, together with diabetes, were associated with increased risk of hospitalisation in participants with and without diabetes. Our study also demonstrated that the presence of diabetes could enhance or attenuate these associations.

Study participants with diabetes were 24% more likely to have a hospital admission for any reason within the year following their recruitment than participants without diabetes and also had more admissions and longer lengths of stay. These findings are variable in relation to a number of previous studies that used different population groups, measures, and follow up periods [4,27,28]. Our study used self-reported information to determine diabetes status while other studies used clinical records including admission data [5,27,28], general practice records and/or registers [4,9,10,27], diabetes clinic data [7], and population registers [5] to identify subjects with diabetes. This to our knowledge is the first time that these associations have been reported in a community dwelling healthy population.

Other studies have reported variable proportions of subjects being admitted. Studies among general practice populations in the UK General Practice Research Database observed a lower rate of admission for all patients with diabetes (33.8 per 100 patient years) [4]. The proportion of patients registered with UK general practices admitted in the year of the study was 25% of those with and 12% without diabetes [9]; and in an Italian linkage study based on citizens registered with health authorities, the proportion admitted was 19.5% and 6.4%% of patients age 40–65 (26.8% and 15.4% for those aged more than 64 year) with and without diabetes respectively [10]. As would be expected, larger proportions of subjects were admitted in studies using longer follow up periods: a New Zealand study using general practice data reported that the proportion admitted across a three year study period was 43.5% and 35.5% among patients with and without diabetes [27]; an Italian study of patients attending a diabetes clinic reported that 55% had at least one admission during 4.5 years of follow-up [7]; and a Finnish study where 50.7% of diabetic patients had at least one hospitalisation during three years of follow-up [5].

Among the participants with diabetes, a diagnostic code indicating a diabetes-related ACSC was recorded for 22% of participants who were admitted. While this finding was generally consistent with other research, some studies report that diabetes related ACSC admissions contribute to a higher percentage of admissions [4,5,27,28]. One explanation for this may be that our community based study population is healthier than the various clinical populations that were employed in comparative studies, that their diabetes is better controlled or they are at an earlier stage of the disease [14]. Other explanations may relate to the definition of diabetes related admissions used [24].

A large number of factors were associated with a self-reported diagnosis of diabetes and with increased risk of hospitalisation in these data. As previously reported, older participants and males in our study were more likely to have a hospital admission recorded independently of diabetes status [4,7,10,29]. Few studies have explored the impact of socioeconomic status or rural residence on admission to hospital. The authors hypothesised that disadvantaged participants and those living in remote locations would have higher rates of hospitalisation reflecting their poorer access to health care services. Previous studies using hospital data have demonstrated increased all cause, ACSC, and diabetes admissions by disadvantage [30,31]. The present study reported mixed results. The risk of admission was lower for participants residing in areas with relative socioeconomic disadvantage and for participants with low educational attainment. These associations were not influenced by diabetes status. Interestingly, the association between household income and hospitalisation was in the other direction with low household income associated with increased risk of hospitalisation. This may reflect a more direct influence on access to health care than the less precise measure of regional disadvantage. We observed a lower risk of hospitalisation for participants residing in outer regional and remote areas compared to those in major cities. This result is different to previous studies using hospital data that suggest much higher rates of hospitalisation for people living outside major population areas compared to those living in major cities [30,31].

Although the overall rates of cigarette smoking in this study are lower than in the general population (17.0% males and 11.8% for females aged 55–65 years) [32], participants who were current or previous smokers were more likely to have an admission. Our observation of an association between obesity and physical inactivity and risk of hospitalisation has been observed elsewhere [7,33]. The observed relatively poor health status of study participants with diabetes and the observed association of health status with increased risk of hospitalisation for all participants has been previously reported [4,7,27,29]. The interesting finding was the attenuation of the association with increased hospitalisation for obesity, high blood pressure and hyperlipidaemia among participants with diabetes and enhancement of hospitalisation admission among those who had been treated for anxiety and depression. These effects were not observed for the wellbeing measures including K-10.

**Figure 1:**
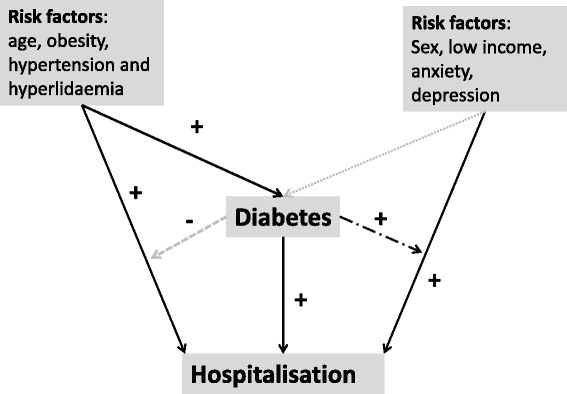
**conceptual framework to explain the impact of diabetes on the associations between other explanatory factors and hospitalisation.** *Coding*: *solid black line*: *positive association*; *dashed*/*dot black line*: *diabetes enhances the association between risk factors and hospitalisation*; *dotted grey line*: *a weak positive association*; *dashed grey line*: *diabetes associated with attenuation of association*.

The differences in the observed strength of the associations between the risk factors examined and risk of hospitalisation for participants with and without diabetes may be explained by the presence of diabetes, the potential for which is suggested in our conceptual framework (Figure 1). These differences were supported by statistically significant interaction terms and changes demonstrated by the ratio of RR. The ratio of RR is a robust means of comparing effect sizes in subgroup analysis and may be more informative than the use of interaction terms. [26] Firstly, male participants were over-represented among participants with diabetes and male participants with diabetes were significantly more likely to be hospitalised than those without diabetes. Not only were a greater proportion of younger participants with diabetes admitted than those without diabetes, the association between age and hospital admission was attenuated for participants with diabetes. This suggests that diabetes may have an “ageing effect” that leads to poorer health at an earlier age for those with diabetes compared to those without diabetes. Once adjusted for age and gender, the patterns of socioeconomic factors influencing the relative rate of hospitalisation were broadly similar (same direction and pattern of factors) for participants with and without diabetes (Table 3). However, participants from rural and remote locations were more likely to have a hospital admission when diabetes was present than when it was not, even though the stratified relative rates indicated that both groups had a lower risk of hospitalisation. While hospitalisation was more common among participants with low income in general, the association between low income and hospitalisation was enhanced among participants with diabetes.

In the stratified analysis, the effects of obesity, hypertension and hyperlipidaemia on the likelihood of hospital admission were reduced in participants with diabetes. One possible interpretation of this result may be that the impact of diabetes on the risk of admission especially for cardiovascular health may be mediated in part by the physiological changes associated with obesity, hypertension and hyperlipidaemia. Thus, when diabetes is accounted for in the modelling, there is little increase in risk of hospitalisation that can be accounted for by these other factors. Further investigation of these relationships is warranted. This finding differs from that of a previous study which showed an enhanced association between hypertension and hospitalisation in people with diabetes [34]. In contrast, the association between male gender, low income, smoking, anxiety or depression and the risk of hospitalisation was enhanced in people with diabetes. This may be due to a variety of factors including synergistic effects of risk factors such as smoking and diabetes on the risk of cardiovascular disease. There may also be synergy in the accessibility of services. For example, the financial burden on patients with diabetes in accessing health services and employment, may exacerbate the effect of low incomes on health and the pattern of health service use. The enhancement of the association between anxiety and depression and hospitalisation has been observed previously [35]. In our study we found that self-report of treatment for these conditions was enhanced among participants with diabetes while there was no modification for K-10 scores, a screening tool for measuring current distress. Poorer health outcomes have also been observed for adults with both diabetes and anxiety and/or depression including higher rates of myocardial infarction [36], and symptom control [37,38].

### Implications for health service provision especially primary care

This study confirms the association between the presence of diabetes and increased rates of hospitalisation. It provides new information on the impact of important risk factors that are associated with both the prevalence of diabetes and higher rates of hospitalisation among people with diabetes. Importantly diabetes mediates some associations between risk factors and hospitalisation with enhancement of some associations and attenuation of others. Although the observed associations between obesity, hypertension and hyperlipidaemia and hospitalisation were reduced after stratification for diabetes, the higher rates of these factors indicates that their control remains important in the prevention of diabetes and of complications and hospitalisation in people with diabetes. The apparent enhancement of the associations between male gender, low income, smoking, and anxiety and depression is and hospitalisation among participants with diabetes is of considerable concern. Males and smokers are lower users of primary care suggesting that a more proactive approach to encourage their use of primary care may be required if rates of admission to hospital are to be lowered. Conversely low income participants and those with anxiety and depression are more frequent users of primary care suggesting that more support for self-management and low cost (to consumer) alternatives to traditional medical care may be more appropriate for these groups. This has direct implications for community education, practice support and quality improvement programs in the development of primary care organisations such as Medicare Locals in Australia, as well as initiatives by diabetes organisations.

### Strengths and limitations

This is one of the largest population based studies of the impact of diabetes on hospitalisation undertaken. Few studies have had the opportunity to link a large population data collection to temporal data in terms of the use of services such as hospitals. A further strength of this study was the availability of comprehensive information about the demographic characteristics, socioeconomic status, lifestyle, and health and wellbeing of participants. These data and identification of the sub-group with diabetes depended on information provided at the baseline survey and we have previously demonstrated that the use of self-report data is an acceptable means of identification [15]. However the linked data used in this study did not include biological information that would have provided confirmatory diagnostic evidence of diabetes status or control of biological indicators of diabetes such as HbA1c levels that have been reported in other studies [7,10,39]. The study could not differentiate between type 1 and type 2 diabetes and was thus not able to explore whether type of diabetes is an important predictor of hospitalisation.. The studies reported were all carried out under different health systems which may have been influenced by different payment systems, admission and discharge policies, and timeliness of data [40].

## Conclusions

This study is one of the few studies published to explore the impact of diabetes on hospitalisation in a large non-clinical population, the 45 and Up Study. Using record linkage, the study demonstrated that participants were 24% more likely to have a hospital admission in the year following recruitment if they reported a diagnosis of diabetes than if they did not. Admitted participants with diabetes had more admissions and longer length of stay than participants without diabetes. Further this study was one of the few to explore the association between a comprehensive set of demographic factors, socioeconomic status, lifestyle and health factors on hospitalisation and associated impact of the presence of a diagnosis of diabetes. Although age and obesity are both major risk factors for diabetes, this study is one of the first to demonstrate attenuation of the associations between both age and obesity and risk of hospitalisation by the presence of diabetes. The increased associations between hospitalisation and other risk factors for hospitalisation such as gender and low income in participants with diabetes may be explained by their synergistic influence on health status and the way services are accessed.
